# Outbreaks of Hemotrophic Mycoplasma Infections in China

**DOI:** 10.3201/eid1507.090174

**Published:** 2009-07

**Authors:** Zhe Hu, Jigang Yin, Kefei Shen, Wei Kang, Qijun Chen

**Affiliations:** Jilin University, Changchun, People’s Republic of China (Z. Hu, J. Yin, K. Shen, W. Kang, Q. Chen); Swedish Institute for Infectious Disease Control and Karolinska Institutet, Stockholm, Sweden (Q. Chen); 1 These authors contributed equally to this article.

**Keywords:** Hemotrophic mycoplasmas, hemoplasmas, China, bacteria, letter

**To the Editor:** Infections caused by hemotrophic mycoplasmas (formerly called eperythrozoonoses ) in animals and humans have been emerging in the People’s Republic of China in recent years. To date, 6 hemotrophic *Mycoplasma* spp. have been identified in rodents and mammals (*1*). *M.* from pigs, *M. wenyonii* from cattle, and *M. ovis* from sheep have been confirmed; the human pathogen, which is most frequently observed in China, has not been genetically identified (*2*). However, the zoonotic potential of the bacteria is evident because the disease is more prevalent in farmers and veterinary doctors, who have frequent close contact with domestic animals, than in other persons (*2*). Vertical transmission from mother to fetus has also been confirmed (*2*). In animals, especially in piglets, the disease is characterized by febrile acute anemia, jaundice, and eventual death resulting from concurrent infection with other microbes (*3*–*6*). Infected humans may be asymptomatic or have various clinical signs, including acute fever, anemia, and severe hemolytic jaundice, especially in infected neonates. Pregnant women and newborns were reported to be more vulnerable to the disease than others and to show more severe clinical signs after infection (*2*).

We conducted an epidemiologic investigation of hemotrophic mycoplasma infections in China by reviewing all reported cases and outbreaks for 1994–2007. Clinical cases for >6 animal species (including pigs, cows, goats, horses, foxes, chickens, and humans) were reported during the period (Table). The number of reported cases varied from year to year. Human infections were confirmed by clinical and laboratory methods (*2*). We reinvestigated blood samples of >600 pigs with previous diagnoses of mycoplasma infection accompanied by clinical signs of fever and jaundice. Slides were made and stained in Giemsa-staining solution. We used light microscopy to look for the presence of *M. suis* on the erythrocyte surface. We also used fluorescence microscopy to look for the microbes by mixing a drop of infected blood with acridine orange solution (0.1 mg/mL). The microbes bound to red blood cells were examined with a confocal microscope. Positive cases were further confirmed by PCR using primers of the small subunit RNA gene sequences. All samples were PCR positive, but PCR sensitivity is higher than sensitivity of acridine orange staining, which is higher than sensitivity of Geimsa staining.

**Table:** Number of reported hemotrophic mycoplasma infections, China, 1994–2007*

Year	Species				
	Human	Cow	Swine	Sheep	Fox
1994	200	NR	NR	NR	NR
1995	331	132	NR	231	NR
1996	1,229	259	147	NR	NR
1997	2,262	69	1,282	126	NR
1998	740	64	127	115	NR
1999	3,861	1,460	397	2,493	954
2000	1,971	2,920	140	NR	371
2001	329	329	7,775	NR	16,697
2002	126	NR	17,068	NR	17,068
2003	880	84	600,033	1,877	31,208
2004	4	625	15,604	206	NR
2005	451	119	27,268	2,916	20
2006	4	75	15,916	536	465
2007	452	3	1,686	53	60

*NR, no record.

Hemotrophic mycoplasma infection is still a neglected zoonotic disease, which poses a threat to public health and the animal industry, especially in China (*2*,*7*). The prevalence of the disease in domestic animals (e.g., pigs) and humans has reached an alarming level (Table). Human infection rates in certain areas in China have been high; for example, in Inner Mongolia, samples collected from 1,529 randomly selected persons during 1994–1996 showed that 35.3% of the local population, 57.0% of local pregnant women, and 100% of newborns of infected mothers were positive for hemotrophic mycoplasma infection (*2*). Infections in animals in China have been recognized since 1995, and the number of cases has been increasing rapidly. For example, >600,000 pigs infected with *M. suis* were reported in 2003 (Table). These infections have had a large economic impact on regions where the infection is endemic (*8*). Infections in other animals, including cows, sheep, and foxes, were also common, indicating a high prevalence of the bacteria in China. However, because of the lack of in vitro cultivation systems that assist in characterizing pathogens, progress in species identification and molecular characterization of these pathogens has been slow. Thus far, names of hemotrophic mycoplasma species have been based on the hosts from which they were identified. Due to the zoonotic nature of these pathogens, more in-depth studies on these microorganisms are needed.
